# Exploring clinical research efficacy of teaching and practicing medical professionals in Pakistan

**DOI:** 10.12669/pjms.37.3.2395

**Published:** 2021

**Authors:** Mubeen Akhtar, Aiman Irfan

**Affiliations:** 1 Mubeen Akhtar, COMSATS University Islamabad (CUI), Park Road, Tarlai Kalan, Islamabad, Pakistan; 2 Aiman Irfan, COMSATS University Islamabad (CUI), Park Road, Tarlai Kalan, Islamabad, Pakistan

**Keywords:** Physicians, Medical education, Research, Competence, Demographic factors

## Abstract

**Objective:**

The current study aimed to explore clinical research efficacy of teaching and practicing medical professionals in Pakistan. The role of socio-demographic factors in this context was also investigated.

**Methods:**

This study using cross-sectional research design was carried out from August to December 2019. A sample of teaching and practicing medical professionals (N=96) was collected through purposive sampling from Islamabad and Rawalpindi. Clinical Research Appraisal Inventory (CRAI) was used along with the demographic datasheet. Research data was analyzed using Statistical Package for Social Sciences (SPSS-21).

**Results:**

The results of the study revealed that teaching and practicing medical professionals feel most competent in ‘collaborating with others’ while the research area in which they feel least competent is ‘securing funds for a study’. It was found that there are significant differences in the research efficacy of teaching and practicing medical professionals with reference to age (p< 0.00), gender (p< 0.01), designation (p< 0.00), number of articles published (p< 0.00), number of articles under review (p< 0.03), number of articles submitted (p< 0.03), and number of funded projects completed (p< 0.02). Satisfaction with salary and number of hours at work per week have no impact on their research efficacy.

**Conclusions:**

Findings have implications for policy makers and medical institutions to promote research skills in teaching and practicing medical professionals.

## INTRODUCTION

The field of medicine can reap enormous benefits from the research; as research plays a very significant role in the analysis of effectiveness and efficiency of health care policies and practices. Moreover, it is also important for the understanding of diseases and diagnosis which in turn influences quality of care provided to the patients.^1^ Self-efficacy is believing in one’s own ability to successfully complete a certain task by organizing the actions required for a person to exhibit a certain result.^2^ Research self-efficacy can be characterized as one’s trust in his/her capacity to effectively complete tasks such as literature reviews and data analysis. Another study stressed that research self-efficacy is crucial for leading a scientific career.^3^ The individual perception of his research self-efficacy affects his participation in research.^4^ Literature suggest that the individual’s research efficacy beliefs depend on his research experiences, available resources, the interest of the investigator and the environment provided to the researcher for research purpose.

Many researchers have identified different barriers that hinder the clinical research; some of them include; lack of funding, lack of experienced clinical researchers, poor collaboration among investigators and poor communication, lack of time, financial barriers and insufficient knowledge about statistical methods.^5^ Researchers argued that the assessment of an individual’s research efficacy will help to find their strengths and weakness in research field. Past research has suggested that there are gender differences in the physicians self-assessed abilities to perform clinical research. Women rated their abilities and skills to perform clinical research lower than the men.^6^

A study found that 85% of general practitioners working in hospitals of Germany has positive attitude towards research activity.^7^ Similarly, another study reported that 90% of general practitioners working in hospitals of United Kingdom acknowledged the significance of research whereas 68% utilized research in patient care.^8^ Literature suggest positive correlation between research efficacy and research productivity.^9^

### Rationale of the Study:

In Pakistan, the competence of teaching and practicing medical professionals and the difficulties they face in conducting research have not been studied thoroughly and the literature available is scarce. A study suggested that local publications have an impact on medical practitioners’ clinical practice.^10^ A research on medical graduates of Karachi found that 59% of participants rated the current research status of Pakistan to be insufficient.^11^ Therefore, to inculcate and promote research environment for teaching and practicing medical professionals it is very essential to find out the areas in which medical practitioner’s research efficacy is high and the aspects in which they are lacking competence. The purpose of this study was also to investigate the differences in research efficacy of physician’s with reference to socio-demographic characteristics (gender, age, designation, years of practice, specialty area, number of hours at work in a week, satisfaction with current pay, number of articles published, number of articles submitted, number of articles under review, number of funded projects completed/ongoing and family influence). This study will provide information regarding the aspects of research process which require training for the teaching and practicing medical professionals to enhance their research involvement.

It was hypothesized that male medical professionals will have higher research efficacy as compared to female medical professionals. Moreover, there will be a significant difference in the research efficacy of teaching and practicing medical professionals with reference to their designation.

## METHODS

This study was a cross-sectional research which was carried out from August 2019 to December 2019 after the ethical approval (Ref. No# CUI-ISB/HUM/ERC-CPA/2020-01 dated Feb. 19, 2020) from departmental Ethics Review Committee (ERC) COMSATS University, Islamabad. The target population of this study was the teaching and practicing medical professionals working in the private and public medical colleges and hospitals of Islamabad and Rawalpindi. A sample of 96 medical professionals (49% female and 51% male) was collected through purposive sampling. For this kind of sampling an inclusion criterion was followed. Only those medical professionals who have a minimum experience of three years of working in the field were included in the sample. From initially contacted 200 participants, 150 met the inclusion criterion which further led to a sample of 96 medical professionals who completed the study. Demographic characteristics of the sample are presented in Table-III.

**Table-I:** Descriptive Statistics and Correlations between Subscales of CRAI (N= 96).

	Variable	1	2	3	4	5	6	7	M (SD)	a (no. of items)
1	CS	1	0.82**	0.88**	0.85**	0.69**	0.60**	0.81**	530.84 (14.88)	0.97 (7)
2	SDA		1	0.72**	0.83**	0.69**	0.80**	0.85**	128.51 (39.16)	0.98 (18)
3	CWO			1	0.81**	0.66**	0.54**	0.68**	47.56 (11.16)	0.96 (6)
4	OS				1	0.82**	0.73**	0.75**	21.79 (6.49)	0.94 (3)
5	PRS					1	0.76**	0.69**	23.32 (5.66)	0.91 (3)
6	FS						1	0.66**	65.32 (28.32)	0.98 (10)
7	RS							1	69.60 (18.50)	0.97 (9)

***Note:*** ***CS:*** Conceptualizing a study, ***SDA:*** Study design and analysis, ***CWO:*** Collaborating with co-workers, ***OS:*** Organizing a study, ***PRS:*** Protecting research subjects, ***FS:*** Funding a study, ***RS:*** Reporting a study,

** p< 0.01

**Table-II:** Ranking of Mean of Means of CRAI-SF Subscales (N= 96).

Rank	Subscales	M	SD
1	Collaborating with others	7.93	1.86
2	Protecting research subject’s privacy and code of conduct	7.77	1.89
3	Reporting a study	7.73	2.06
4	Conceptualizing a study	7.69	2.12
5	Organizing a study	7.26	2.16
6	Study design and data analysis	7.14	2.18
7	Funding a study	6.53	2.83

**Table-III:** Demographic Characteristics of Sample and Group Differences on CRAI (N= 96).

Variables	Category	f	%	M	SD	t/F	p
Gender	Female	47	49.0	380.06	110.38	2.66	0.01
	Male	49	51.0	438.63	105.18		
Age	< 40	65	67.7	381.35	104.42	7.77	0.00
	40-50	20	20.8	477.55	101.55		
	> 50	11	11.5	456.09	105.86		
Designation	Senior Medical officer	29	30.2	336.14	115.27	5.24	0.00
	PG Trainee	22	22.9	419.41	81.67		
	Consultant	08	8.3	447.88	96.79		
	HOD	09	9.4	462.0	100.40		
	Senior Lecturer/ Demonstrator	07	7.3	387.29	47.46		
	Assistant Prof.	11	11.5	440.55	98.80		
	Associate Prof.	10	10.4	508.30	104.24		
Years of practice	< 10 years	58	60.4	389.97	114.82	2.73	0.07
	10-20 years	32	33.3	446.19	100.95		
	> 20 years	06	6.2	410.00	90.81		
No. of hours at work per week	< 20	05	5.2	392.00	135.76	0.42	0.66
	20-40	49	51.0	401.90	108.56		
	> 40	42	43.8	421.50	113.04		
Satisfied with salary	Yes	36	37.5	415.08	105.61	0.35	0.73
	No	60	62.5	406.88	115.12		
No. of articles published	Nil	44	45.8	372.73	110.47	9.16	0.00
	1-5	32	33.3	410.34	104.98		
	> 5	20	20.8	491.25	77.52		
No. of articles under review	Nil	53	55.2	387.70	108.66	2.22	0.03
	1-5	43	44.8	437.40	109.23		
No. of articles submitted	Nil	69	71.9	394.12	112.54	2.24	0.03
	1-5	27	28.1	450.42	100.20		
No. of projects completed	Nil	74	77.1	397.14	105.08	2.11	0.02
	1-5	22	22.9	453.09	122.40		
No. of projects ongoing	Nil	84	87.5	402.29	109.14	1.81	0.07
	1-5	12	12.5	463.67	115.05		
Anyone in family whose job require research work	Yes	43	44.8	424.79	128.03	1.18	0.24
	No	53	55.2	397.92	94.89		

### Clinical Research Appraisal Inventory:

**(**CRAI), was used to assess clinical research self-efficacy of teaching and practicing medical professionals.^12^ It was chosen because of being a comprehensive and reliable tool to assess the degree of confidence in performing common clinical research tasks. Eller and colleagues developed the short form of the scale which is a 56-item scale consisting of six domains of research efficacy including conceptualizing a study, study design and analysis, collaborating with coworkers, organizing a study, protecting research subjects, funding a study, and reporting a study.^13^ Each item is scored on an 11-point scale, where 0 signifies no confidence and 10 signifies total confidence in a skill relevant to clinical research. Total scores are obtained by summing numeric responses to each item. The higher the score, the higher the respondent’s research self-efficacy. Internal consistency (Chronbach alpha) of subscales and total scale ranged from 0.84 to 0.98.^13^ The scale was pilot tested on a small sample of 15 teaching and practicing medical professionals to make sure the feasibility of the study in terms of time requirement to provide required information, understandability of instructions and relevance of all domains of scale for the medical professionals. The results of the pilot test required no changes in the scale.

A demographic data sheet was used to collect information regarding gender, age, designation, years of practice, specialty area, number of hours at work in a week, satisfied with the current salary, number of articles (published, submitted and under review) number of funded projects (ongoing and completed) and family influence (in terms of any support in research endeavor because of being their involvement in research).

Data was collected from hospitals and medical colleges with the approval of the respective authorities. The purpose of study was explained to medical professionals before delivering questionnaire. Written informed consent was sought from them. SPSS version 21 was used for the analysis of data. Correlational analysis, t-test and one-way ANOVA were computed.

## RESULTS

CRAI-SF has good reliability coefficient as shown by Cronbach’s Alpha coefficient (see Table-I). The subscales have very good reliability ranging from 0.97 to 0.90. Correlations between CRAI-SF subscales is also presented in Table-I. It shows significant positive correlation between the subscales.

The ranking of mean of means of CRAI-SF subscales is shown in Table-II. It shows that the teaching and practicing medical professionals feel most confident/competent in collaborating with others (*M=*7.93), whereas the area in which medical professionals feel least competent is securing funds for a study (*M=* 6.53).

Result of t-test, presented in Table-III, showed significant difference between the mean of female and male medical professionals on total CRAI score (p=0.01) as hypothesized. Male medical professionals have higher research efficacy as compared to female teaching and practicing professionals. Further analysis revealed that men have significant high scores on subscales of study design and data analysis (*p*=0.01), organizing a study (*p*=0.01), and finding funding for a study (*p*=0.00).

In accordance with the hypothesis there are significant differences between the mean scores of teaching and practicing medical professionals belonging to different designations (Table-III). The mean score of teaching and practicing medical professionals having higher designations is greater than the mean score of medical professionals having lower designations. In the current study, the mean of associate professor is highest followed by HOD’s and assistant professors (*p*=0.00).

Significant differences were found concerning age of the medical professionals. Medical professionals who fall in the age range of 40-50 years have significantly higher mean scores on CRAI-SF (*p*=0.00) as compared to other groups. Further analysis revealed this group has significant higher score on ‘conceptualizing a study’ (*p*= 0.00), ‘study design and analysis’ (*p*=0.00), and ‘funding a study’ (*p*=0.03). However, teaching and practicing medical professionals above 50 years of age have significantly high mean score on ‘collaborating with others’ (*p*= 0.00), and ‘organizing a study’ (*p*=0.01).

Number of articles (published, under-review, submitted) has an impact on research efficacy of teaching and practicing medical professionals (Table-III). It was found that the medical professionals who have five or more published articles have significantly higher mean score than the ones having no publication or fewer publication (*p*=0.00). Significant differences were found when comparing the mean of those medical professionals whose articles are under review and those whose articles are not under review in overall CRAI score (*p*=0.03) and three of its subscales; study design and data analysis (*p*= 0.04), organizing a study (*p*=0.03) and reporting a study (*p*=0.00). In all these subscales the mean of medical professionals whose articles are under review is greater than the medical professionals belonging from the other group. The mean of those teaching and practicing medical professionals who have submitted articles is greater than those who haven’t in the subscale of conceptualizing a study (*p*=0.03), study design and data analysis (*p*=0.02), reporting the study (*p*=0.03) and in overall CRAI score (*p*=0.03).

Significant differences were found when comparing the mean of teaching and practicing medical professionals who have completed funded projects and those who haven’t completed any funded project (Table-III). Those medical professionals who have completed the funded projects have higher mean score in subscales of funding a study (*p*=0.00) and in overall CRAI score (*p*=0.02). The mean of those medical professionals who are currently involved in a funded project is greater than those who aren’t involved in the subscale of ‘funding a study’ (*p*=0.01).

There are no significant differences in research efficacy of teaching and practicing medical professionals concerning number of hours per week, satisfaction with current salary, number of years of experience, and family influence.

## DISCUSSION

According to the findings of this research, the hypothesis concerning the gender differences in clinical research efficacy is supported by the data of the study. The results of the study showed that the men have more confidence in their abilities to perform research related tasks as compared to women. In all the subscales of CRAI-SF the mean scores of male teaching and practicing medical professionals were greater than female teaching and practicing medical professionals. Existing literature also shows similar results. A past study also found significant gender differences in research performance.^14^ They found that men publish more articles as compared to women. However, the explanation that the researchers gave for this difference is that, women are concerned with the quality rather than the quantity.^15^ Female researches write fewer articles but are cited more than their male counterparts. These results corroborate our finding as there is a relationship between research efficacy and research productivity.^16^ Besides, responsibilities of family life, parenthood and residential work results in less time for the enhancement of their research careers.^15^ Furthermore, past research has suggested that scholarly bolster and mentoring provided to women is less as compared to men. This may be one of the reasons of their low research efficacy than men as reliance on mentor help the careers grow.^17^

There is significant difference in research efficacy of teaching and practicing medical professionals holding various designations, as hypothesized. The results of our study showed that research efficacy of associate professors and assistant professors is significantly high as compared to other designations. It shows that senior position is associated with better rating of research abilities. This is in accordance with available international literature. The designation of the teaching and practicing medical professionals plays a vital role in the rating of their confidence to carry out various research related tasks.^5^ This can be attributed to the fact that research publication is essential for these medical professionals as a part of their career. Certain numbers of publications are essential for appointments and promotions. The research efficacy of PG trainees was better than senior medical officers, this may be due to the requirement of publication of two research papers as a part of their training.

As far as the impact of age on research efficacy is concerned, the results of our study showed that teaching and practicing medical professionals in the age range of 40-50 years have higher efficacy as compared to those younger and older than them. Past research has documented that productivity increases with age, the older researchers are more productive, and their impact is greater as compared to younger researchers. The productivity of researchers is at its peak when they are in the middle of their careers as compared to those who are at the start. Current study found that teaching and practicing medical professionals aged 50 and above rated themselves more in ‘collaborating with others’ than other age groups. Literature suggested that the influence of old researchers after 50 is mostly linked to building a strong group, including younger researchers.^18^

Number of articles (published, under-review, submitted) and number of projects (completed and ongoing) have an association with research efficacy of teaching and practicing medical professionals. Previous studies have suggested strong positive correlation between number of articles published/submitted with the research efficacy of individuals. Research efficacy is affected by research engagement. According to a study number of articles published is one of the predictors of research self-efficacy.^19^

Furthermore, our study didn’t find any significant differences in research efficacy of teaching and practicing medical professionals based on satisfaction with pay, number of hours at work per week, no of years of experience, family influence and specialty area. The reason of this non- significant difference may be that research is intrinsic interest. If the person is really interested in something, then his efficacy wouldn’t be affected by extrinsic variables. A past research found no effect of pay on the intrinsic interests of individuals.^20^ The personal motivation of an individual also plays a significant role in increased research performance.^5^

Findings of the study revealed the research areas in which teaching and practicing medical professionals feel most competent/confident and least competent/confident. The results indicated that medical professionals feel most competent in ‘collaborating with others. This is in accordance with the literature which suggests that medical is a profession which is facilitated by collaboration between professionals.^21^ It is essential for medical practitioners to reach at specific diagnosis and to get better patient outcomes. This may be the reason of high competency in collaborating with others as it is a part of their job. The present study further showed that the research area in which teaching and practicing medical professionals feel least competent/confident is ‘securing funds for a study’. This may be attributed to the shortage of funds for scientific research and related competitiveness for available grants in Pakistan. Also, in the area of spending on research Pakistan is ranked low.^22^

### Limitations of the study:

Firstly, the study sampled the research participants from a single city which may have limit the generalizability of the findings. Secondly, response rate was low as the questionnaire was slightly long which made participants reluctant to respond. Future research may benefit by using a time efficient yet reliable data collection tool.

## CONCLUSION

The findings of the study showed that research efficacy among teaching and practicing medical professionals is affected by gender, age, designation, no. of articles (published, under review and submitted), and funded projects (completed and ongoing). However, satisfaction with current pay, number of hours at work per week, and family influence have no impact on their research efficacy. It also revealed that teaching and practicing medical professionals feel most competent in collaborating with others while the research area in which they feel least competent is securing funds for a study.

### Recommendations:

This study has implications for policy makers and institutions. To promote research among medical professionals, policy makers should increase the budget allocated for research. At academic level, research awareness should be raised among students. Workshops should be organized to increase research attitude among residents and medical practitioners. The engagement of young medical practitioners in research activity will give a boost to local medical community. Efforts are needed to encourage women to participate in research endeavors and enhance their research efficacy.

## References

[1] Munir N, Bolderston A (2009). Perceptions and attitudes toward conducting research:A nuclear medicine student perspective. J Med Imaging Radiati Sci.

[2] Bandura A (1997). Self-Efficacy. The exercise of control.

[3] Forester M, Kahn JH, Hesson-McInnis MS (2004). Factor structures of three measures of research self-efficacy. J Career Assess.

[4] Bieschke KJ (2006). Research self-efficacy beliefs and research outcome expectations:Implications for developing scientifically minded psychologists. J Career Assess.

[5] Khalaf AJ, Aljowder AI, Buhamaid MJ, Alansari MF, Jassim GA (2019). Attitudes and barriers towards conducting research amongst primary care physicians in Bahrain:a cross-sectional study. BMC Family Pract.

[6] Bakken LL, Sheridan J, Carnes M (2003). Gender differences among physician–scientists in self-assessed abilities to perform clinical research. Acad Med.

[7] Rosemann T, Szecsenyi J (2004). General practitioners'attitudes towards research in primary care:qualitative results of a cross sectional study. BMC Family Pract.

[8] Robinson G, Gould M (2000). What are the attitudes of general practitioners towards research?. Br J Gen Pract.

[9] Kahn JH, Scott NA (1997). Predictors of research productivity and science-related career goals among counseling psychology doctoral students. Couns Psychol.

[10] Aslam F, Qayyum MA, Mahmud H, Qasim R, Haque IU (2004). Attitudes and practices of postgraduate medical trainees towards research--a snapshot from Faisalabad. J Pakistan Med Assoc.

[11] Ejaz K, Shamim MS, Shamim MS, Hussain SA (2011). Involvement of medical students and fresh medical graduates of Karachi, Pakistan in research. J Pak Med Assoc.

[12] Mullikin EA, Bakken LL, Betz NE (2007). Assessing research self-efficacy in physician-scientists:the clinical research appraisal inventory. J Career Assess.

[13] Eller LS, Lev EL, Bakken LL (2014). Development and testing of the clinical research appraisal inventory-short form. J Nursing Meas.

[14] Cole J.R, Zuckerman H (1987). Marriage, motherhood and research performance in science. Sci Am.

[15] Symonds MR, Gemmell NJ, Braisher TL, Gorringe KL, Elgar MA (2006). Gender differences in publication output:towards an unbiased metric of research performance. PLoS One.

[16] Pasupathy R, Siwatu KO (2014). An investigation of research self-efficacy beliefs and research productivity among faculty members at an emerging research university in the USA. Higher Educ Res Dev.

[17] Van Balen B, Van Arensbergen P, Van Der Weijden I, Van Den Besselaar P (2012). Determinants of success in academic careers. Higher Educ Policy.

[18] Liang L, Guo Y, Davis M (2002). Collaborative patterns and age structures in Chinese publications. Scientometrics.

[19] Landino RA, Owen SV (1988). Self-efficacy in university faculty. J Vocat Behav.

[20] Fang M, Gerhart B (2012). Does pay for performance diminish intrinsic interest?. Int J Human Res Manag.

[21] Braithwaite J, Westbrook M (2005). Rethinking clinical organisational structures:an attitude survey of doctors, nurses and allied health staff in clinical directorates. J Health Serv Res Policy.

[22] Shahid J Is research and development still not a priority for the government. Dawn.

